# Smoking prevalence, attitudes and associated factors among students in health-related Departments of Community College in rural Yemen

**DOI:** 10.18332/tid/92547

**Published:** 2018-07-03

**Authors:** Abdulsalam M.A. Nasser, Bassam A.M. Salah, Luba T. Regassa, Abdulrakeeb A.S. Alhakimy, Xinping Zhang

**Affiliations:** 1 School of Medicine and Health Management, Tongji Medical Collage, Wuhan, China; 2 Institute of Urology, Department of Urology, The First Affiliated Hospital of China Medical University, Shenyang, China

**Keywords:** smoking, attitudes, associated factors, rural area, Yemen

## Abstract

**INTRODUCTION:**

Tobacco smoking is a global concern and tobacco use is rising among the youth in Arab countries, such as Yemen, especially among university students. This study aims to examine the prevalence, attitudes and associated factors of smoking among college students in the rural area of Hajja, Yemen.

**METHODS:**

A cross-sectional descriptive study was conducted at a community college in the Hajja province between August and December 2016. The data were collected from three health related departments (Nursing, Laboratory, and Assistant doctor). A questionnaire based on the Global Health Professional Survey and the Global Youth Tobacco Survey was answered by 380 randomly selected students.

**RESULTS:**

Logistic regression analyses indicated that sex (OR=0.4, 95% CI: 0.2–0.8, p<0.05), family income (OR=2.0, 95% CI: 1.2–3.3, p<0.05), and residence (OR=0.2, 95% CI: 0.1–0.5, p<0.001), were statistically significant predictors of smoking. Smoking for peer pressure, recreation and proving manhood were all found to be highly significant (p<0.001). The results also revealed that smokers had more negative attitudes towards allowing children to smoke in the future and allowing smoking in the household (p<0.001).

**CONCLUSIONS:**

Compared to similar studies on the general population, the prevalence of smoking among college students in the rural area was lower. An indication of a growth in tobacco use can be concluded, accordingly, the need for anti-smoking programmes to prevent students from initiating smoking is required.

## INTRODUCTION

Smoking is a leading cause of preventable morbidity and mortality worldwide. Tobacco smoking causes annually 6 million deaths worldwide and is projected to exceed 8 million by 2030, according to statistics from the World Health Organization (WHO)^1^. Smoking was identified as the most important cause of preventable morbidity and premature death^2^. This ratio is estimated to increase to 10 million in 20–30 years. Due to tobacco use, the morbidity rate is 70% in developing countries, and these countries are the ones in which problems due to epidemic tobacco use are mostly seen^3-5^.

The WHO estimates the number of smoking individuals as 1.1 billion, globally. A total of 700 million male smokers are located in developing countries, and 47% of men and 12% of women smoke a total of 6 trillion cigarettes a year worldwide^5^. According to the Global Youth Tobacco Survey (GYTS) conducted in 2001, in five Arab countries in the Middle East, approximately 10% of youths (13–15 years of age) use different kinds of tobacco products^6^.

On the other hand, a recent study on smoking among Arab-American adolescents found that 26.6% of the studied sample smoked waterpipes^7^, emphasizing the increasing trend of this form of smoking among Arab youths. In Lebanon and Jordan, studies have reported that the prevalence of waterpipe smoking was more common than cigarette smoking among university students^8,9^.

A household survey conducted by the WHO in some Arab countries in 2012 stated that the prevalence of smoking any tobacco product among those aged ≥15 years in 2009 was 24% and 1% in the KSA, 46% and 31% in Lebanon, 47% and 6% in Jordan, 35% and 4% in Kuwait, 34% and 8% in Bahrain, 58% and 5% in Tunisia, and 35% and 11% in Yemen, among males and females, respectively^10^. Based on the Global Adult Tobacco Survey (GATS) conducted on the population aged ≥15 years in Qatar in 2013, the results indicated that the prevalence of waterpipe use was 4.9% and 1.6% among males and females, respectively^11^. On the other hand, a report from the WHO on the global tobacco epidemic, conducted on the population aged 15 years or more in Yemen in 2017 concluded that the estimated prevalence of smoking was 18.7% among both sexes^12^.

However, the prevalence of smoking, especially among university students, is largely unknown in many of these Arab countries, including Yemen, and to the best of our knowledge, no student-based survey has been conducted on the prevalence of smoking and its associated factors in the rural area of Hajja, Yemen.

The goal of the study is to determine the ratio, attitudes and associated factors of smoking among college students in the rural area of Hajja, Yemen.

## METHODS

A cross-sectional descriptive study was done during the winter course of the 2016 academic year upon the approval from the review board of the community college located in the Hajja province of Yemen. A self-administered, anonymous questionnaire about smoking, attitudes and associated factors was completed by college students in three different health-related departments of the community college (Nursing, Laboratory, and Assistant doctor).

### Sample

The community college has seven departments. All three health-related departments namely Nursing, Laboratory, and Assistant doctor were purposively selected. We aimed to recruit >35% from the total 1093 students registered at these three health-related departments in 2016, based on the total number of students in each department of not equal size; the sample from each department was chosen to represent proportionately the total number of students in that department. Hence, about 400 (36.6%) students were randomly selected and invited to take part in the survey. All 400 students approached agreed to complete the questionnaire, yielding a response rate of 100%. Due to incorrect filling in, 20 questionnaires were discarded and a total of 380 (response rate 95%) were analysed.

### Data collection

Questionnaire forms were delivered to students in their classrooms during breaks and were collected as soon as they were completed. Students had been informed that questionnaire participation was voluntary, their identity would not be recorded on the form, all the data in the questionnaire would be used for research purposes, and no penalty would be applied against non-participants.

The questions gathered information about basic demographic properties and issues regarding tobacco usage, including participants’ points of view, smoking behaviours, habits and attitudes. The questionnaire was in Arabic and English, and derived from models based on the Global Youth Tobacco Survey^13^ and Global Health Professional Survey^14^.

The first items (sociodemographic profile) covered sex, age, type of college, marital status, residence, grade, family economic situation and smoking style (both cigarette and waterpipe). This was followed by questions about attitudes and beliefs concerning smoking (smoking or not, smoking reasons, allowing children to smoke in the future, prohibiting smoking at home, and prohibiting it in public areas).

### Definition

Smoking status was established in accordance with the WHO criteria for cigarette smoking and the criteria set by Maziak et al. for waterpipe smoking^15,16^. Smokers in this survey referred to subjects who at the time of the survey smoked either regularly one cigarette/day or more, or one waterpipe/week. Non-smokers were defined as students who never smoked one cigarette in their lifetime and who did not smoke at all during the time of the survey.

According to World Bank’s country and lending categorizations (2016), Yemen^17^ was in the lower-middle-income economies with a Gross National Income (GNI) per capita per year of US$ 1006–3955. Therefore, the family income item was categorized into low (US$ < 100/month), middle (US$ >100–200/month) and high (US$ >200–300/month).

### Statistical analysis

The computer package SPSS version 20 was used for data analysis. Data were presented as numbers and percentages, and were compared using a chi-squared test. Logistic regression was also applied to investigate the relationship between tobacco usage and the socio-economic factors such as sex, age, marital status, study level, residence, income and college. The results based on p-value varied between highly significant, significant and not significant. Significance was considered for p< 0.05.

## RESULTS

### Characteristics of the samples

This study included a total of 380 students; 234 (61.6%) were males and 146 were females (38.4%). More than (76.1%) were aged from 18 to 24 years. The mean age was 21.43 ± 2.27 years. Most of the participants were in the department of Nursing (43.4%), followed by the department of Laboratory (32.4%) and the department of Assistant doctor (24.2%). Most students were married (84.2%) and living with their parents (73.9%). About 54.5% of the students were from low-income families.

The prevalence of smoking was higher among students who were living in dormitories than those who were living with their families (23.2% vs 8.5%, p<0.001). Similarly, it was higher among students from low-income families compared to those from average and high-income ones (15.9%, 10.8% and 3.2%, respectively, p<0.05) (Table 1).

**Table 1:** Smoking status of the study sample based on the demographic characteristics in the rural area (N=380)

*Characteristics*	*Total n*	*Per cent (%)*	*Smokers n (%)*	*Non-smokers n (%)*	*χ2*	*p*
**Age (years)**						
<18	49	12.9	5 (10.2)	44 (89.8)	0.688	0.747
18–24	289	76.1	38 (13.1)	251 (86.9)		
Over 24	42	11.1	4 (9.5)	38 (90.5)		
**College Department**						
Nursing	165	43.4	23 (13.9)	142 (86.1)	0.677	0.719
Laboratory	123	32.4	14 (11.4)	109 (88.6)		
Assistant Doctor	92	24.2	10 (10.9)	82 (89.1)		
**Marital status**						
Married	320	84.2	39 (12.2)	281 (87.8)	0.061	0.831
Single	60	15.8	8 (13.3)	52 (86.7)		
**Study level**						
First-year	269	70.8	28 (10.4)	241 (89.6)	4.289	0.118
Second-year	54	14.2	11 (20.4)	43 (79.6)		
Third-year	57	15.0	8 (14.0)	49 (86.0)		
**Residence**						
With family	281	73.9	24 (8.5)	257 (91.5)	14.578	<0.000
Dormitory	99	26.1	23 (23.2)	76 (76.8)		
**Family monthly income**						
Low	207	54.5	33 (15.9)	174 (84.1)	7.469	0. 022
Average	111	29.2	12 (10.8)	99 (89.2)		
High	62	16.3	2 (3.2)	60 (96.8)		

The total prevalence of smoking among students was 12.4% (cigarettes 7.4% and waterpipe 5.0%). No student reported to be a dual user of cigarettes and waterpipe. The prevalence of cigarette smoking among males was 10.3% and 2.7% for females, while the prevalence of waterpipe smoking among males was 0.0% and 13.0% among females (p<0.001).

Table 2 shows factors that were associated with being a smoker. The covariates examined were gender (female vs male), age (<18, 18–24 years and over 24 years), study level (first, second and third year), marital status (single vs married), family income (low, average, high), residence (dormitory vs family home) and college departments (Nursing,Laboratory, Assistant doctors). There was a strong statistically significant association of smoking with residence (p<0.001). Sex (p<0.05) and family income (p<0.05) were statistically associated with smoking. There was no significant association between the risk of smoking and age, marital status, College departments or study level. The logistic regression analysis indicated that females were less likely to be smokers (OR=0.4, 95% CI: 0.2–0.8) and the respondents who lived with their family were also found less likely to be smokers (OR=0.2, 95% CI: 0.1–0.5). On the other hand, the lower family income appeared to be a risk factor of being a smoker (OR=2.0, 95% CI: 1.2–3.3).

**Table 2:** Relationship between demographic factors and smoking among college students in the rural area

*Variable*	*β*	*SE*	*Wald–χ2*	*p*	*OR ( 95% CI)*
Sex	-0.883	0.345	6.554	0.010	0.4 (0.2–0.8)
Age	0.165	0.368	0.201	0.654	1.2 (0.6–2.4)
Marital status	0.235	0.466	0.254	0.614	1.3 (0.5–3.2)
Study level	-0.258	0.214	1.455	0.228	0.8 (0.5–1.2)
Residence	-1.453	0.351	17.187	0.000	0.2 (0.1–0.5)
Family income	-0.676	0.268	6.349	0.012	2.0 (1.2–3.3)
College departments	0.117	0.218	0.287	0.592	1.1 (0.7–1.7)

β: estimated coefficient, SE: standard error, OR: odds ratio, CI: confidence interval.

Table 3 reveals the attitudes of smokers and non-smokers towards different smoking styles and the prohibiting of indoor or outdoor smoking areas. Smokers had a positive attitude towards smoking compared to non-smokers (100.0% vs 0.00%, p<0.001). Analysing the reasons why students tend to smoke revealed a highly significant difference (p<0.001); 15.7% of smokers versus 84.3% of non-smokers thought that students smoke for recreational reasons, while 23.3% of smokers and 76.7% of non-smokers believed that students may smoke because of peer pressure. Smokers appeared to have more negative attitudes towards allowing children to smoke in the future compared to non-smokers (77.8% and 22.2%, respectively, p<0.001). Most of the smokers would allow smoking in their household compared to non-smokers (64.3% vs 35.7%, p<0.001).

**Table 3:** Attitudes and beliefs concerning smoking among college students in rural areas (N=380)

*Items*	*Total n (%)*		*Smokers n (%)*	*Non-smokers n (%)*	*χ2*	*p*
**Do you agree with smoking behaviour?**						
Yes	18	4.7	18 (100.0)	0 (00.0)	Fisher’s exact	<0.000
No	362	95.3	29 (8.0)	333 (92.0)		
**Why do students smoke?**						
Recreation	121	31.8	19 (15.7)	102 (84.3)	34.171	<0.000
Proving manhood	52	13.7	0 (00.0)	52 (100.0)		
Peer pressure	120	31.6	28 (23.3)	92 (76.7)		
Other reasons	87	22.9	0 (00.0)	87 (100.0)		
**Would you allow smoking in your household?**						
Yes	42	11.1	27 (64.3)	15 (35.7)	117.426	<0.000
**No**	338	88.9	20 (5.9)	318 (94.1)		
**Would you allow your children to Smoke in the future?**						
Yes	18	4.7	14 (77.8)	4 (22.2)	Fisher’s exact	<0.000
No	362	95.3	33 (9.1)	329 (90.9)		
**Do you agree with banning smoking in public areas?**						
Yes	308	81.1	34 (11.0)	274 (89.0)	2.651	0.113
No	72	18.9	13 (18.1)	59 (81.9)		

## DISCUSSION

In this study, the percentage of the prevalence of smoking among college students in rural areas was found to be 12.4%. This prevalence was higher than the prevalence rate in some equivalent studies conducted on ages ranging from 18 to 28 years in Iran (9.8%)^18^.

On the other hand, the prevalence rate was lower compared with the results from similar studies in Arab countries conducted among health-related students, including 46% in Kuwait^19^, 17.2% in Jordan^20^, 46.7% in Egypt^21^ and 26.3% in Lebanon^22^.

In terms of smoking prevalence among males and females, the conclusions from this study are that cigarette smoking was higher for males than females, 10.3% vs 2.7%, a result in agreement with previous studies that reported on some Arab countries, such as the KSA (32.7% vs 5.9%)^23^ and the UAE (84.6% vs 15.4%)^24^.

The study revealed that a greater proportion of females were waterpipe smokers compared to male smokers (13.0% vs 0.0%). Similarly, a previous study conducted in Jordan concluded that the prevalence of waterpipe smokers was higher among females than males (88.6% vs 36.6%)^25^. A Palestinian study also revealed a high rate of waterpipe smoking among female university students^26^. The prevalence of waterpipe smoking is predominant among female students. This reflects the fact that cigarette smoking is being replaced by waterpipe use, which is considered as an aspect of a modern lifestyle or prestige among the youth of the Eastern Mediterranean region^27,28^. This may be attributed to the fact that cigarette smoking is regarded as inappropriate behaviour for women in Yemeni culture, unlike waterpipe smoking which is quite tolerated and socially accepted and seen in common social interactions, such as gatherings and household meetings in Arab societies, giving waterpipe smoking the privilege of being socially accepted as part of the cultural heritage.

Based on statistical analysis, smoking prevalence was highly influenced by residence, as the rate was 23.2% for students living in dormitories and 8.5% for those living with their families, in agreement with the findings of a similar study in Syria^29^. Gfroerer et al.^30^ also came to the conclusion that college students in the US who lived with their parents were less likely to smoke than students who did not. The reason may be that students who live with their families are less socially, emotionally and psychologically stressed than those who live abroad in dormitories and hence more susceptible to smoking. Another reason may be friends, as indicated also by previous studies on medical students in Japan and Albania; these studies reported that friends were the most important factor associated with smoking behaviours^31,32^.

In the current study, the prevalence rate of smoking was significantly affected by family income, that is, the percentage of smokers among students from low income families was higher than those from average and high income ones (15.9%, 10.8%, and 3.2%, respectively); this finding was found to be in conformity with the world health survey, which stated that the poorest men were over 2.5 times more likely to smoke than the richest men in numerous countries^33^. A possible reason also could be that students with a lower socioeconomic status had more physical, psychosocial and emotional problems.

Peer pressure and recreation were found to be the main reasons for students becoming involved in smoking, while ‘proving manhood’ was a major reason for non-smokers’ attitudes, according to this study; similar findings were reported by previous studies^34^.

In this study, the attitudes of smoking and non-smoking students towards smoking were contrasted; smokers were more tolerant of smoking at home and would be less likely to put pressure on their children not to smoke. Smokers were also less enthusiastic about prohibiting smoking in public places. Moreover, they had more negative attitudes with regard to allowing children to smoke in the future and allowing smoking in the household. Similar findings were obtained by a study in Palestine^34^.

The reasons for smoking identified in this study should be taken into account in the adoption of antismoking programmes, which could make them more effective and better able to influence the attitudes and behaviours of smokers. The study provides implications for policy makers. The ministry of education and higher education should apply anti-smoking programmes in all universities. Programmes need to be established that involve teenagers and youths as educators, and they should be supplied with correct and suitable information about the health consequences of smoking to educate the community. In addition, the media can assist in publicizing the anti-smoking messages to the whole population in Yemen.

### Strengths and limitations

This study was the first student-based survey on the prevalence of smoking and its associated factors on students of health-related departments at a community college in the Hajja province of Yemen. It demonstrated the prevalence of smoking among students and associated factors in a rural area of Yemen. The study covered three health-related departments out of seven in the community college in a rural area. Hence, the results can be generalized to similar areas with similar social characteristics.

We believe our study has some limitations. First, since it was a cross-sectional study, the reported findings might be affected by reporting bias and the data may reflect respondents’ subjective perceptions. Second, since the studied sample size of students from the rural area of the Hajja province was not relatively large, the applicability of the results to urban areas may be limited. Third, we did not examine the effects of cigarette and waterpipe smoking on rural areas. Hence, further studies are needed to clarify the effects of all kinds of smoking in the rural areas of Yemen.

## CONCLUSIONS

The prevalence of smoking among college students in the rural area of the current study was found to be lower than that released by the WHO report^12^ of 2017.

This study also revealed that male students were cigarette smokers, while females were waterpipe smokers. The prevalence of waterpipe users among females, as opposed to males, is a matter of concern. Factors such as sex, residence and family income were strongly associated with smoking prevalence. Youth from both genders have an ongoing attraction to cigarettes and waterpipes. An indication of a growth in tobacco use can be concluded. This necessitates the development of smoking cessation programmes, awareness raising and the promotion of anti-smoking attitudes in schools, colleges and universities in the region. Adopting anti-smoking programmes to prevent the harmful effects of smoking are urgently required in these colleges. Information on tobacco control policies should be widely disseminated.
